# Exploring the importance and preference of sugar feeding behaviour of malaria vectors in sugar plantations of southern Malawi

**DOI:** 10.1371/journal.pone.0344351

**Published:** 2026-03-06

**Authors:** Kennedy Zembere, Sylvester Coleman, James Chirombo, Rex Mbewe, Julie-Anne Tangena

**Affiliations:** 1 Vector Biology Research Group, Malawi Liverpool Wellcome Programme, Blantyre, Malawi; 2 Department of Vector Biology, Liverpool School of Tropical Medicine, Liverpool, United Kingdom; 3 Department of Clinical Sciences, Liverpool School of Tropical Medicine, Liverpool, United Kingdom; 4 Entomology Section, Malaria Alert Centre, Kamuzu University of Health Sciences, Blantyre, Malawi; Clinton Health Access Initiative, UNITED STATES OF AMERICA

## Abstract

**Background:**

Reliable tools are needed to control opportunistic outdoor biting and resting malaria vectors that remain beyond the reach of indoor targeted interventions. The attractive targeted sugar baits (ATSBs) have demonstrated effectiveness in some settings but have shown limited impact in other areas, in part due to differences in mosquito species’ preferences and the presence of competing natural sugar sources. We evaluated the sugar-feeding preferences of *Anopheles gambiae* in Chikwawa, southern Malawi, to inform context specific sugar-based vector control interventions.

**Methods:**

Using three collection tools, CDC Light traps; Prokopack aspirator and the barrier screen, we collected 187 adult anophelines from the Illovo sugar plantations. Collected mosquitoes were subjected to cold anthrone tests in the laboratory to assess the presence of plant sugars in their gut. Additionally, 810 adult *Anopheles gambiae* s.l., reared in the insectary from wild caught larvae, were exposed in an olfactory-driven choice experiment to identify the most attractive available sugar source in the area. Sugar sources included guavas, melon, bananas, mango, marula and sugarcane.

**Results:**

Over 40% (n = 74) of the collected *Anopheles* mosquitoes- including *An. gambiae* s.l., *An. funestus*, *An. coustani* and *An. tenebrous* were found to have fed on natural sugar sources. For the sugar attractiveness tests for *An. gambiae* s.l., guava was found to be twice as attractive (IRR = 1.97, 95% CI: 1.49–2.62, p < 0.001) as sugarcane (our reference fruit), followed by banana (IRR = 1.68, 95% CI: 1.26–2.24, P < 0.001), then mango, and melon (IRR = 1.49, 95% CI: 1.11–2.01, P = 0.008) and (IRR = 1.45, 95% CI: 1.08–1.96, P = 0.014) respectively.

**Conclusion:**

Sugar feeding is a key activity for *Anopheles* mosquitoes and presents a potential target for control. Understanding local sugar source preferences will help tailor novel mosquito control intervention strategies such as the ATSBs to specific ecological contexts.

## Introduction

Insecticide-treated bed nets (ITNs), indoor residual spraying (IRS), and malaria case management using artemisinin-based combination therapies (ACTs) have contributed to worldwide malaria transmission reduction [1–3]. Between 2000 and 2025, malaria deaths reduced by over 50% worldwide [2,4]. In the same period, over 1.57 billion cases of malaria were averted [2] with most of this success attributed to ITN use [2,4]. Despite this success, malaria remains one of the leading causes of mortality in Africa, even in areas with good vector control coverage [5]. Since 2015, we have seen malaria cases increase by about 23% [6]. This has, in part, been attributed to the increased level of insecticide resistance and behavioural shifts in vectors and the focus of vector control tools on only indoor biting and resting vectors [7,8]. Thus, ITNs and IRS use alone cannot achieve complete malaria control and elimination, as opportunistic outdoor biting and resting vectors remain beyond their reach, leading to sustained residual malaria transmission [7,8].

Alternative control tools have been proposed for use in both indoor and outdoor environments to complement existing vector control tools. One promising approach is the attractive targeted sugar bait (ATSB). These traps use a combination of fruits or flower scents (baits), oral toxins and sugar to attract mosquitoes [9,10], leveraging their need for glucose meals [11]. Unlike traditional vector control tools that target indoor resting and biting mosquitoes, the ATSBs target the entire vector population, including males, and have the potential to reduce the density of both indoor and outdoor mosquitoes [12]. Despite promising results from earlier entomological trials in Mali and Israel, recent large-scale trials in Kenya and Zambia [13,14] failed to demonstrate significant epidemiological or entomological impacts of ATSBs, raising critical questions about the context-specific efficacy of ATSBs and the underlying sugar-feeding behaviours of vector populations. Further studies from different ecological settings have thus been recommended [15].

Effective implementation of ATSBs and other tools that exploit sugar feeding behaviour requires thorough understanding of the role of sugar sources in malaria vector ecology, the attractiveness of different sugar sources, and overall mosquito sugar-feeding behaviour. Previous studies have shown that *Anopheles gambiae* s.l. exhibits strong preference for specific sugar sources [16,17]. These findings emphasize that local mosquito sugar-feeding behaviour, especially in sugar-rich environments like southern Malawi, can determine ATSB success. While the question of which blood hosts mosquitoes prefer has been extensively studied, in Malawi and across Africa, little is known about the sugar sources most commonly foraged by malaria vectors [17]. This study evaluated the anopheline mosquito sugar feeding behaviour as a baseline to understand the importance and preference of sugar sources, providing key insights to guide implementation of vector control tools that exploit sugar feeding behaviour.

## Methodology

### Study area

The study was conducted within the Illovo sugar estate in Nchalo, Chikwawa (− 16.1869; 34.8805), the largest sugarcane producer in Malawi. More details regarding the study area have been discussed elsewhere [18] but in summary, the estate sits within a malaria endemic region in the Shire Valley of Chikwawa district, southern Malawi. *Anopheles arabiensis* and *An. funestus* are the two major vectors in the study area [18]. With over 13,000 hectares of sugarcane plantations, the study area relies heavily on extensive irrigation systems during the dry season, thereby sustaining mosquito breeding all year round.

### Testing the presence of sugar in the gut of wild-caught *Anopheles* mosquitoes

**Fig 1:**
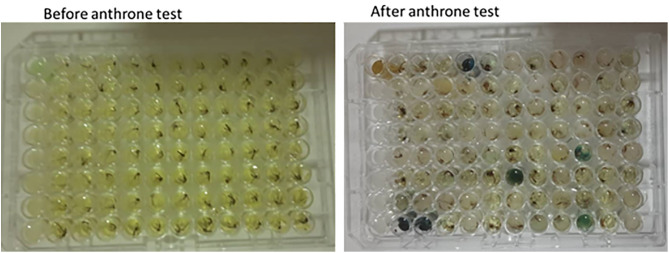
Cold anthrone test. A. Mosquitoes in ELISA plates before cold anthrone test. B. Mosquitoes that fed on plant sugars changed colour to blue-green after a cold anthrone test.

We evaluated the proportion of anopheline mosquitoes that fed on sugar using the cold anthrone test, which detects fructose in the mosquito gut [19]. Adult mosquitoes were collected in August 2023 for a period of four weeks, with two consecutive days of collection each week. Mosquitoes were collected between 6 pm to 6 am using CDC light traps, Prokopack aspirators and barrier screens outdoors next to sugarcane farming plots, focusing on areas near putative breeding sites and surrounding bushes to target newly emerged mosquitoes. It has been shown previously that newly emerged and nulliparous mosquitoes are more likely to feed on sugar sources than older mosquitoes that have previously blood fed or are parous [20]. Thus, newly emerged mosquitoes are a key target for sugar feeding exploitation methods. Barrier screens were also set near sugar plantations and near bushes and breeding sites that were close to human habitations to target host seeking mosquitoes that fly towards the households to seek blood or those leaving houses looking for places to oviposit. All mosquitoes were identified morphologically to species level using standard identification keys [21]. Within 2 hours of collection, all *Anopheles* mosquitoes collected (both males and females) were tested for the presence of fructose using the cold anthrone test [19] before the sugar was degraded/digested inside the mosquito gut [22]. A 500 ml cold anthrone yellow reagent was prepared by adding 1 gram of anthrone powder to a mixture of 140 ml of distilled water and 360 ml concentrated sulphuric acid in a 1000 ml screw cap bottle. To test for the presence of fructose in mosquito guts, mosquitoes were singly placed in ELISA plate wells (Fig 1). Well A1 was left for positive control with a known sugar (table sugar-sucrose) and B1-H1 were left for negative control with distilled water only.

Whole individual mosquitoes were placed in each well and crushed using separate pestles to release the fructose. A total of 200 ul of cold anthrone reagent was dispensed into each well. The plate was then covered and incubated for 45 minutes at room temperature. After incubation, the results were scored based on the colouration, where yellow (no colour change) was interpreted as negative (no sugar) and blue-green colour change indicated a positive result (presence of fructose (Fig 1).

### Sugar choice experiments

Mosquito larvae were collected in the field and reared in the laboratory for the olfactory-driven behavioural assay. Within the Chikwawa sugar plantation area, random sampling was done, stratified by proximity to sugar plantation, to select areas for larval surveillance. Stratification was done to maximise larvae presence as it has previously been shown that pollen-rich standing pools of water, associated with the sugar plantations, are potential mosquito oviposition sites and larval habitats for gravid *An. arabiensis* [23]. We used probability sampling methods to ensure that all the breeding sites had a chance of being selected. Thus, all breeding sites had equal chances of being selected if they were near sugarcane plantations. We sampled 10 larval habitats in total. Habitats generally comprised of relatively small to medium-sized irrigation channels within the Illovo sugarcane plantations. Collected mosquito larvae were reared under insectary conditions [24] and emerged adults (3–5 days old) were used for sugar choice experiments. All larvae collected in the wild turned out to be *An. gambiae* s.l*.* after rearing in the insectary. Thus, only *An. gambiae* s.l. were used for the sugar source comparison study.

Different plants, identified as potential sugar sources based on previous studies [22,25] were used in the choice-experiment studies. Additional criteria were based on plant prevalence in the study area and possible importance as a sugar source during the study period as they were in season. Sugar sources used in the experiments were guava (*Psidium guajava)*, melon (*Cucumis melo)*, banana (*Musa sp*), mango (*Mangifera indica*), marula *(Sclerocarya birrea)* and sugarcane (*Saccharum officinarum*). They were crushed to maximize the release of volatile compounds and odours.

**Fig 2:**
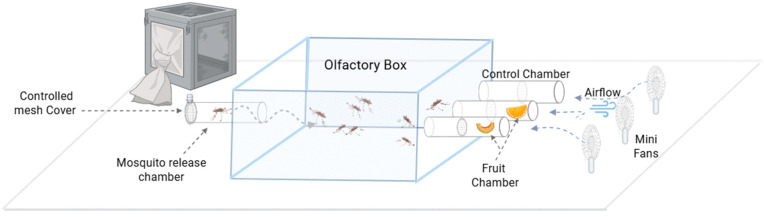
The olfactory driven behavioural assay box: An experimental set up which includes two types of fruits and a control. Starved *An. gambiae* s.l. from a cage were released through the release chamber into the olfactory box using a mouth aspirator. Release chamber was closed using a controlled mesh cover to hold mosquitoes. Mini fans were used to push scents into the box.

**Fig 3:**
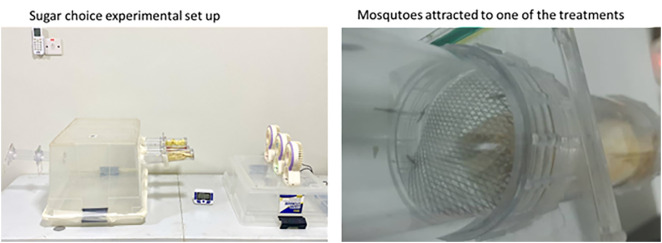
Sugar choice experiments. A. Mosquito sugar choice experimental set up. B. Mosquitoes attracted in a tube with one of the fruit treatments. Number of mosquitoes attracted by each treatment was determined by counting how many mosquitoes entered each tube with the different treatments compared to the control.

A total of 810 wild *Anopheles gambiae* s.l. reared from larvae were used for the sugar attractiveness experiments in the laboratory at the Malawi Liverpool Wellcome Chikwawa site. Before exposure, mosquitoes were starved for 24 hours but given only water to keep them hydrated. Mosquito attractiveness to six naturally available sugar sources were determined compared to a control (distilled water) using an olfactory driven behavioural assay box (Figs 2 and 3). The assay was locally designed based on [23,26] with modifications to simultaneously compare the attractiveness of three different compounds in laboratory conditions.

Mosquito attractiveness experiments were conducted using nine experimental set-ups (S1 Table), each consisting of three experiments where sugar sources were rotated every 30 minutes to minimize positional bias. Thirty fresh mosquitoes were released per experimental rotation, and a Latin square design was used to control for treatment effects and positional variations (S1 Table). A new cohort of mosquitoes was used in each setup, and previously used mosquitoes were aspirated from the chamber, killed with ethanol, and discarded. This was to prevent carryover effect, as previously used mosquitoes exposed to the same odours could have a greater potential to habituate and learn, thereby increasing attractiveness bias.

Each set-up included two fruit types and a control (e.g., mango, guava, and a control in tubes A, B, and C, respectively) (Figs 2 and 3). In the laboratory, mosquitoes were introduced into a box olfactometer via a release chamber equipped with a mesh cover and sliding glass for controlled entry. Chambers were approximately 15 cm apart, and mini fans facilitated airflow, directing fruit/plant odours toward the mosquitoes.

To reduce positional bias, treatment positions were switched twice across three experimental runs: first, with mango, guava, and control in tubes A, B, and C; then, with guava, control, and mango; and finally, with control, mango, and guava. The experiment was repeated for all set-ups. Between trials, acetone was used to clean the tubes to eliminate residual odours. Mosquito responses were recorded based on the number entering each treatment tube compared to the control.

#### Sample size calculation.

Sample size was determined pragmatically based on feasibility and operational considerations rather than through a formal a priori sample size calculation. To maximise the number of adult mosquitoes collected by the traps for the cold-anthrone experiments and the number of larvae collected from the larval habitats for choice experiments, the sample sizes were evaluated post hoc. As a result, the sample size achieved reflects field conditions and logistical constraints rather than a prespecified power requirement, and the findings should therefore be interpreted with appropriate caution, particularly with respect to statistical precision and the strength of inferential conclusions.

### Statistical analysis

To assess the presence of sugars, we fitted a logistic regression model to estimate the presence of fructose in the wild caught anopheline mosquitoes. Due to the nested nature of the data, we included the trap as the random effect to investigate any possible differences between the traps.

To determine the preference for potential sugar sources for *An. gambiae s.l.,* we first performed an unadjusted analysis to compare the performance of the different treatments (sugar sources) in attracting mosquitoes. To achieve this, we performed the Kruskal-Wallis test for comparing the median number of mosquitoes across all the treatments. We then performed multiple comparison using Bonferroni correction to compare the number of mosquitoes caught by different pairs of treatments. Then, in the adjusted analysis, we fitted a negative binomial regression model with the number of mosquitoes caught by each method as the response variable and treatment as a fixed effect. The negative binomial model was chosen to account for the possible effect of overdispersion, which occurs when the variance exceeds the mean. The treatment fixed effect allowed us to compare the different treatments. For the negative binomial regression model, we calculated the incident rate ratio (IRR) to measure the covariate effects, as it provides an interpretable measure of the multiplicative change in the expected count of mosquitoes per unit change in the predictor variables. For example, an IRR of 1.5 for a given predictor indicates that a one-unit increase in that variable is associated with a 50% increase in the expected count of mosquitoes, holding other variables constant.

We fitted two models. In the first model, we did not account for the study design, thus leading to a simple negative binomial generalised linear model (GLM) without random effects. In the second model, we fitted a negative binomial mixed-effects model to account for the study design by letting the experiments be the random intercept. Model comparison was done through the Akaike Information Criteria (AIC) to select the best-fitting model for further inference. We then performed model diagnostics on the selected model.

Let $Y_{i}$ be the number of mosquitoes caught in treatment i. We assumed that $Y_{i}$ follows a negative binomial distribution with mean $\mu_{i}$, allowing for overdispersion in the count data. The mixed-effects regression model was specified as


$$\text{log}\left(\mu_{i}\right)=\alpha +X_{i}\beta +Z_{i}u$$

Where α is the intercept, $X_{i}$ is a design matrix of fixed-effect predictors, β is the corresponding vector of coefficients and $Z_{i}$ is the design matrix for random effects with associated random intercepts u. The random effects were assumed to be normally distributed with mean zero and constant variance. The fixed-effects GLM excluded the random-effects term ${𝐙}_{i}𝐮$. All analyses were done in the R environment for statistical computing**.**

### Ethical approval and consent to participate

The study was conducted in Malawi, with ethical approval from the College of Medicine Research and Ethics Committee (COMREC) (P.05/23/4101). Prior to the study commencement, support was obtained from the District Health Officer of Chikwawa and officials from the Illovo sugar estate. No human participants were recruited in this study, and thus, no informed consent was required.

## Inclusivity in global research

Additional information regarding the ethical, cultural, and scientific considerations specific to inclusivity in global research is included in the Supporting Information (S1 Checklist).

## Results

### Exploring the presence of sugars in wild-caught *Anopheles* mosquitoes

A total of 181 out of the 187 *Anopheles* mosquitoes were successfully tested, of which only 2 were males. Four *Anopheles* species were identified: *An. gambiae* s.l. *and An. funestus,* which are the primary malaria vectors in the study area; and *An. coustani* and *An. tenebrosus*, which are secondary vectors. Overall, over 40% (n = 74) of the anopheline mosquitoes collected were positive for sugar, indicating they had fed on sugar sources. Logistic regression was used to model the probability that an individual mosquito tested positive for fructose (Table 1). There was no statistically significant difference in the presence of sugar among the four different *Anopheles* species. *An. gambiae* s.l., for example, exhibited an odds ratio (OR) of 1.75 (95% CI: 0.33–12.94) for sugar ingestion compared to *An. tenebrosus*, indicating a potentially, but statistically non-significant, higher likelihood of sugar consumption. In contrast, *An. funestus* (OR = 0.95, 95% CI: 0.16–7.70) and *An. coustani* (OR = 0.74, 95% CI: 0.11–6.17) showed lower odds of sugar ingestion relative to *An. tenebrosus*.

**Table 1:** Logistic regression model estimating the presence of fructose in the wild caught anopheline mosquitoes.

Predictors	Number tested	Number positive	OR	CI	P
Intercept			0.50	0.07–2.56	0.42
*An. tenebrosus*	6	2	*Comparator*		
*An. coustani*	26	7	0.74	0.11–6.17	0.75
*An. funestus*	31	10	0.95	0.16–7.70	0.96
*An. gambiae* s.l.	118	55	1.75	0.33–12.94	0.53

#### Proportion of sugar feeding mosquitoes per trap type.

**Fig 4:**
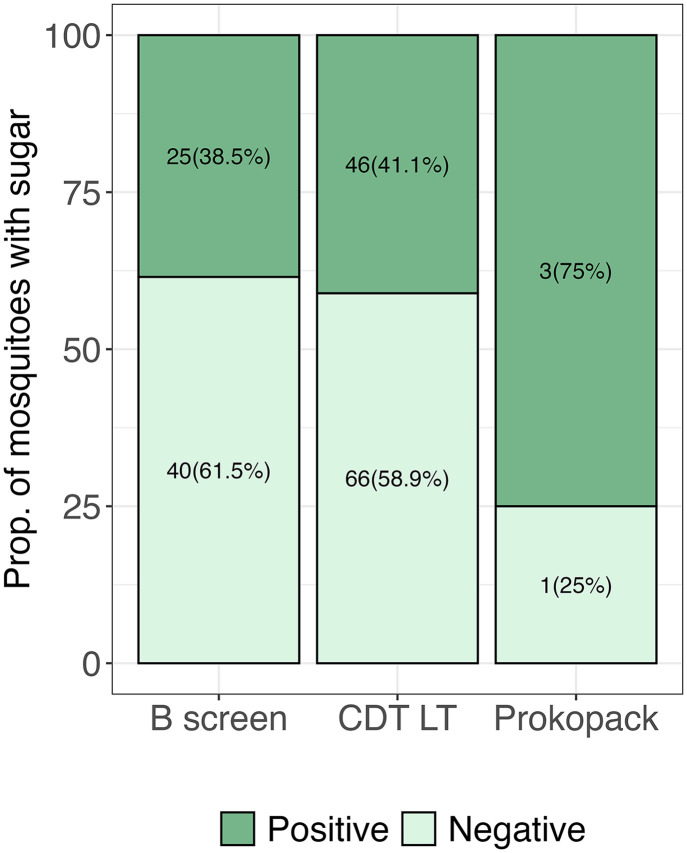
Proportion of mosquitoes that fed on plant sugars by trap type used.

Here, we present the performance of the mosquito collection tools (CDC light traps, Prokopack aspirator, and Barrier screen) used to collect sugar-fed mosquitoes in a natural setting. From our findings, the CDC light trap and the barrier screen collected similar proportions of mosquitoes that fed on sugar (Fig 4). The results showed that 41.1% (n = 46) of *Anopheles* mosquitoes collected by the CDC LT tested positive for sugar while 38.5% (n = 25) collected by the barrier screen tested positive. For the barrier screens, it was interesting to note that mosquito abundance varied depending on the side of the barrier screen. Generally, over 93% (n = 61) of the mosquitoes were collected from the side of the barrier screen facing the breeding sites while only a few (n = 4) mosquitoes were collected from the other side of the barrier screen that faced people’s dwelling places. The Prokopack aspirator collected very few mosquitoes in general compared to the other collection methods (n = 4). However, proportionally, the Prokopack had the highest percentage of mosquitoes that fed on plant sugars (75%); thus, n = 3 out of the n = 4 mosquitoes had sugar in the gut (Fig 4).

#### Determining the attractiveness of fruits to Anopheles gambiae s.l. mosquitoes.

**Fig 5:**
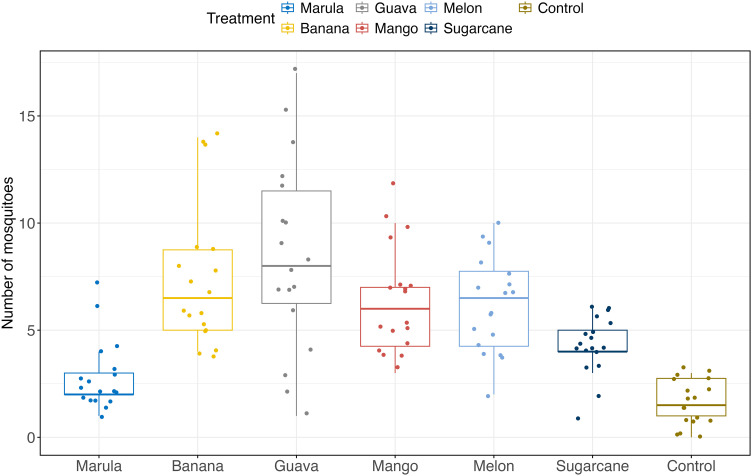
Attractiveness of mosquitoes to treatments in all experiments (experiments 1, 2 and 3) across the 9 setups.

Fig 5 summarizes the average number of *An. gambiae* s.l. attracted to each of the local sugar sources mosquitoes were exposed to. In general, the results show that mosquitoes were most attracted to guava with marula being the least attractive fruit.

A Kruskal-Wallis test revealed significant differences in mosquito attractiveness to the different sugar sources (p < 0.01). Pairwise comparisons demonstrated that marula differed significantly from banana, guava, mango, and melon (p < 0.001), but not from sugarcane. Attractiveness of mosquitoes to banana did not significantly differ from guava, mango, or melon, but was significantly different from sugarcane (p = 0.02). We further fitted a GLM to compare fruit attractiveness to mosquitoes, taking into account the nested random effects. Our analysis revealed a significantly higher incidence of mosquito attraction for all tested fruits compared to sugarcane (as a reference) (Table 2). Guava was the most attractive fruit, attracting mosquitoes at approximately twice the rate as sugarcane (IRR = 1.97, 95% CI: 1.49–2.62). Banana attracted mosquitoes at a rate 1.68 times higher than sugarcane (IRR = 1.68, 95% CI: 1.26–2.24). Mango and melon also demonstrated significantly higher mosquito attraction rates compared to sugarcane (IRR = 1.49, 95% CI: 1.11–2.01) and (IRR = 1.45, 95% CI: 1.08–1.96) respectively. As expected, the control demonstrated significantly lower compared to the reference (IRR = 0.14, 95% CI: 0.09–0.22) (Table 2). Comparing the attractiveness across treatments, mosquito attractiveness varied significantly between treatments (p < 0.0001). Overall, guava was shown to be the most attractive treatment with the highest median mosquito count, while marula and sugarcane were the least attractive (Fig 5).

**Table 2:** Comparison of fruit attractiveness to mosquitoes.

Predictors	N	IRR	CI		p	
Intercept		1.07	0.85–1.35		0.568	
Sugarcane	77	*Comparator*				
Marula	51	1.32	0.92–1.90		0.130	
Banana	135	1.68	1.26–2.24		**<0.001**	
Guava	152	1.97	1.49–2.62		**<0.001**	
Mango	115	1.49	1.11–2.01		**0.008**	
Melon	112	1.45	1.08–1.96		**0.014**	
Control	29	0.14	0.09–0.22		**<0.001**	

## Discussion

We assessed the prevalence of sugar feeding in wild anopheline mosquitoes and the attractiveness of local fruits to *An. gambiae* s.l. in a laboratory setting. For the first time in Malawi, our study revealed potential plant sugar sources that are attractive to *Anopheles* mosquitoes.

Using the cold anthrone test, our study confirmed the presence of sugar in the guts of both primary and secondary malaria vectors, suggesting that they actively utilise sugar sources in the environment. Compared to a study in Kenya, where about 15.7% of the collected mosquitoes had fed on sugar [22], we found that over 40% of mosquitoes had sugar in their guts. This difference may be due to variations in mosquito species, local vegetation, environmental conditions, sampling settings, or mosquito sampling methods. However, our sample size was small, and these results should be considered preliminary and require further studies to confirm the observed patterns.

Different trapping methods were used to maximise collections and to capture mosquitoes at various physiological stages (e.g., host-seeking, resting, or dispersing). We found no significant differences in the proportion of sugar-fed mosquitoes across collection methods, as both barrier screens and CDC light traps yielded similar proportions. Although the Prokopack collected the highest proportion of sugar-fed mosquitoes, the lower number of mosquitoes collected by this method increases the likelihood that the observed differences were due to chance.

The differences in trap performance were likely due to several factors. The CDC light traps, for example, primarily attract host-seeking females, while barrier screens are more effective at intercepting host-seeking or dispersing female mosquitoes. On the other hand, the Prokopack is designed to collect resting mosquitoes of both sexes [27,28]. This could in part explain why all males were collected by the Prokopack, although only a few males were collected.

The limited number of males collected during the study could be due to the timing of collections (6 pm–6 am). Previous studies had associated increased sugar feeding from as early as 5 pm until around 7 am in the morning [21,28], although this was not only specific to males. Secondly, the low numbers could be attributed to inefficiencies in trapping methods, which are biased against female mosquito host-seeking behaviour [29]. Thus, male-based sampling tools such as swarm nets that target swarming males should be considered in future studies [30]. Nevertheless, males are obligate sugar feeders, exclusively requiring sugar for their energy needs [31]. Therefore, exploiting mosquito sugar feeding behaviour, allows us to target both sexes as part of integrated mosquito control [14,24,32,33]. If properly implemented, this may help suppress the mosquito population by strategically placing ATSBs near swarming sites to reduce their survival and mating ability [24,32,34]. However, the small sample size in this study prevents definitive conclusions about sex-specific sugar-feeding behaviour. Future studies should consider including trapping tools and collection times that can maximise the collection of both sexes.

These results from the cold anthrone experiment provide insight into sugar-feeding activities by the primary and secondary malaria vectors in the study area that can be exploited in designing sugar-based control tools such as the ATSBs. In settings with diverse natural sugar plants, for instance, abundant sugar sources may serve as alternative sugar meals, reducing mosquito contact with tools such as ATSBs [21,35]. Previous studies have attributed reduced ATSBs efficacy to the availability of diverse natural sugar plants that compete with them [36,37], highlighting the need for studies like the current one to provide evidence that mosquitoes are highly attracted to natural sugars. This may help to quantify the degree of competition ATSBs may face from natural sugar sources in a particular setting.

Having determined the mosquito sugar feeding behaviour, we further explored the attractiveness of different plant sugar sources to *An. gambiae* s.l. Using the box olfactometer behavioural bioassay, we found there was a significant attraction of *An. gambiae* s.l. to all sugar sources tested compared to the control. Although all six sugar sources tested were attractive, there were variations in their attractiveness to *Anopheles gambiae* s.l., signifying that different plants may have differences in their importance as sugar sources. Elsewhere, it was also shown that *An. gambiae* s.l. demonstrates selectivity in its foraging activity towards plant species [25,38]. In our study, guava, followed by banana seemed to be the most attractive while marula and sugarcane were the least attractive. However, bananas and guavas exhibited a greater variability in attractiveness, as indicated by their wider interquartile ranges (Fig 5), suggesting the presence of potential outliers in the data. Consistent with a previous study in Mali, guava was reported as the most attractive fruit to *An. gambiae* s.l. compared to other fruits (banana, sugarcane, melon) [39]. In contrast to our findings the authors found bananas to be less attractive to *An. gambiae* s.l. while sugarcane was reported as one of the most attractive plants in the same study in Mali [39]. The differences between our observations and the study in Mali could be due to differences in mosquito species. The predominant species identified in the current study was *An. gambiae* s.l. Although mosquito molecular identification was not performed in this study, recent studies suggest that *An. arabiensis* is likely the dominant species, where it accounts for over 80% of *An. gambiae* s.l. population in Chikwawa district [18,40–42]. However, *An. gambiae* s.s. makes up about 86% of the *An. gambiae* s.l. found in the study area in Mali [25]. Whereas mango was attractive to mosquitoes but less so than guava in the current study, a study in Kenya found that mango was the most attractive, although it used mango flowers [37] rather than fruits. The use of fruits in our experiments might account for the differences between our study and the Kenyan study.

There may be several explanations for the poor performance of sugarcane observed in our study area compared to the study in Mali. One possible, though unverified, explanation is variation in sugarcane genotypes. Some studies have shown that the genotype and environment affect productivity and overall sugar content of sugarcane [43]. Furthermore, sugar content of sugarcane can be influenced by the ages, whether they are early or mid-late maturing sugarcane varieties [44]. These differences could further lead to differences in attractiveness to different mosquitoes. However, the present study did not characterise the sugarcane variants. Additionally, no specific varieties were mentioned in the Mali study; therefore, the claim about the differences remains speculative and should be cautiously interpreted. Additional studies are required to compare known sugarcane varieties under similar ecological conditions.

Sugar source preference can vary between species and seasons. Previous studies have shown that female mosquitoes exhibit preferences for specific plant sugars, with some plants being over 130 times more attractive than others [45–47]. However, in the absence of preferred plants, mosquitoes can switch to feeding on less attractive but available alternatives [48]. Therefore, plants that are abundant or whose flowering period coincides with the peak density of mosquito populations could be explored for further evaluations. Despite the seemingly low attractiveness of sugarcane in our study, the widespread and perennial presence of sugarcane from the Illovo farms – one of the largest sugarcane plantations in southern Africa [18] still makes it an important sugar source. Sugarcane from the plantation may serve as an alternative sugar source that sustains the vector populations when the most attractive plants are scarce, thus undermining control efforts. This underscores the need for further research to quantify the contribution of this perennial sugar source to vector bionomics.

In general, the study showed that there is a clear preference for certain sugar sources by the *An. gambiae* s.l. tested, highlighting an opportunity to tailor tools to local preferences and lure them away from natural sugar sources. The current study thus, adds to the evidence from other studies that suggests that natural sugar foraging behaviour in mosquitoes could be exploited for control [12,22,25,49,33]. The survival rate of mosquitoes is a very important factor in the mosquito vectorial capacity [50–53]. Sugar feeding plays a crucial role in mosquito survival. Thus, studies on the effects of interventions on mosquito survival rates (e.g., sugar-based control tools) are important and have previously been recommended. Such studies, however, require baseline data to understand local mosquito sugar-feeding bionomics.

The data from this exploratory study could be significant for designing and deploying future studies and for guiding the selection of potential plant sugars to use in sugar baits when implementing sugar-based mosquito control tools in Malawi. The results from the sugar choice experiments highlight the need to consider ecological contexts and local floral landscapes when designing and planning sugar-based interventions. Selecting highly attractive sugar sources can improve the efficacy of sugar-based interventions such as ATSBs [37,54]. Incorporating guava-based attractants or synthetic blends that mimic volatiles associated with guava should be explored as a strategy to optimise ATSB efficacy in the study area. However, since our sugar attractiveness study was laboratory-based, there is a need for field-based studies to replicate these findings in the natural world. Additionally, the observation from the cold anthrone test, that a considerable proportion of the mosquitoes entering the community had already fed on natural sugar, as inferred from the barrier screen collections, has implications for the deployment of sugar-baited trap stations. We hypothesise that deploying multiple bait stations around breeding sites or at the point of entry into communities might improve its efficiency than currently reported. While the practicality of this may be questioned, it may be worth exploring in separate studies.

Another factor worth investigating is the role of visual stimuli in mosquito attraction to sugar sources. Previous research [25] has highlighted the importance of visual cues, showing that mosquitoes may be drawn to a plant based on its presentation. Another study, aimed at assessing sugar feeding behaviour of *Anopheles* mosquitoes in a setting with diverse natural sugar sources, is ongoing in the study area and will be reported separately.

### Limitations

One of the limitations of this study was the low number of mosquitoes collected, which may have been influenced by seasonality and the timing of the study. The study was conducted at the beginning of the dry season and did not align with peak mosquito abundance. Seasonal impacts on the abundance of *Anopheles* mosquitoes have been reported previously [55,56]. Additionally, the effect of seasonality on sugar feeding has been reported elsewhere, where reduced sugar availability during the dry season may increase biting rate despite decreased vector abundance [57]. This is because vectors have fewer alternative feeding options, which consequently increases the human biting rate and, consequently, vectorial capacity [57]. Further studies, including both seasons, could increase opportunities to capture more mosquitoes and enable comparisons across seasons, possibly also increasing the proportion of sugar-fed mosquitoes captured due to increased sugar sources during the rainy season. Another limitation of this study is that no molecular identification was performed for An. *gambiae* s.l to distinguish between *An. gambiae* sibling species. Due to their differences in feeding and ecological behaviours, our interpretation of the findings may be limited, as these species have varying sugar-feeding preferences, as shown elsewhere [16,17]. However, recent studies from the study area indicate that over 90% of the *Anopheles* mosquitoes within the *An. gambiae* complex is *An. arabiensis* [18,40–42]. Thus, it is likely that *An. arabiensis* accounted for the majority of the mosquitoes collected in this study. Nonetheless, molecular analysis would be ideal to confirm this, and therefore, the observed sugar-feeding preferences should only be interpreted with regard to *An. gambiae* s.l. Future studies should therefore incorporate molecular identifications to account for species-specific sugar-feeding behaviour.

Another limitation relates to the relatively small number of mosquitoes captured using some of the collection methods. This is reflected in the wide confidence intervals observed in the logistic regression analyses, which indicate limited precision of the estimated effects. As a result, these findings should be interpreted as exploratory rather than confirmatory. Future studies with larger and more balanced sample sizes across collection methods and seasons will be important for improving precision and for assessing the robustness and generalisability of the observed patterns.

Additionally, we did not analyse plant DNA from the wild-caught mosquitoes. Thus, genetic sequencing of collected mosquitoes could complement the cold anthrone tests and sugar choice experiments. This could have confirmed whether mosquitoes had fed on the plants identified as attractive in behavioural bioassays. Future studies should incorporate molecular analysis of mosquito gut contents to verify plant-feeding behaviour under field conditions.

While the composition of plant sugar may influence mosquito attraction, our study did not analyse the sugar content of the plants used. Further research should investigate chemical composition of these sugars for better understanding of their role in mosquito attraction. A recent study [57] has demonstrated the potential to identify plant hosts from nectar metabolites inside mosquito guts, and a similar study would be valuable in Malawi.

## Conclusion

Sugar feeding is a key activity for *Anopheles* mosquitoes and presents a potential target for control efforts. In this setting with abundant sugarcane, guava was identified as the most attractive sugar source for *An. gambiae* s.l., followed by banana, mango, and melon, with sugarcane being the least attractive. Understanding local sugar source preferences can help tailor novel intervention strategies to specific environmental contexts. While the current laboratory study provides baseline evidence of potential sugar preferences in Malawi, future studies in a field setting are recommended to extend these results and enhance validity and replicability across diverse conditions. Additionally, future studies should consider comparing flowers and other plant materials besides fruits. This will inform evidence-based selection of sugar sources when designing effective ATSB implementation.

## Supporting information

S1 Table Latin square design for the sugar choice experiments. (DOCX)

S2 Table Cold anthrone test data set. (CSV)

S3 Table Sugar preferences data set. (CSV)

S1 Checklist Inclusivity in global research. (DOCX)

S1 File Graphical Abstract. (TIF)
