# Supplementary material for: Exploring the importance and preference of sugar feeding behaviour of malaria vectors in sugar plantations of southern Malawi
Source: PLoS One. 2026 Mar 6;21(3):e0344351. doi: 10.1371/journal.pone.0344351 (PMC12965689; doi:10.1371/journal.pone.0344351)
Supplement: S1 Table — (DOCX) [file pone.0344351.s001.docx]

**Latin square design for the sugar choice experiments**

| **Experiment** | **Treatment** | | |
| --- | --- | --- | --- |
|  | **1** | **2** | **3** |
| **Experiment 1** | Guava | Sugarcane | Control |
| Run 1 |  |  |  |
| Run 2 | Sugarcane | Control | Guava |
| Run 3 | Control | Guava | Sugarcane |
| **Experiment 2** | Banana | Control | Malura |
| Run 1 |  |  |  |
| Run 2 | Control | Malura | Banana |
| Run 3 | Malura | Banana | Control |
| **Experiment 3** | Melon | Control | Guava |
| Run 1 |  |  |  |
| Run2 | Control | Guava | Melon |
| Run 3 | Guava | Melon | Control |
| **Experiment 4** | Sugarcane | Control | Melon |
| Run 1 |  |  |  |
| Run 2 | Control | Melon | Sugarcane |
| Run 3 | Melon | Sugarcane | Control |
| **Experiment 5** | Banana | Mango | Control |
| Run 1 |  |  |  |
| Run 2 | Control | Banana | Mango |
| Run 3 | Mango | Control | Banana |
| **Experiment 6** | Malura | Mango | Control |
| Run 1 |  |  |  |
| Run 2 | Control | Malura | Mango |
| Run 3 | Mango | Control | Malura |
| **Experiment 7** | Sugarcane | Mango | control |
| Run 1 |  |  |  |
| Run 2 | Control | Sugarcane | Mango |
| Run 3 | Mango | Control | Sugarcane |
| **Experiment 8** | Malura | Melon | Control |
| Run 1 |  |  |  |
| Run 2 | Control | Malura | Melon |
| Run 3 | Melon | Control | Malura |
| **Experiment 9** | Banana | Guava | Control |
| Run 1 |  |  |  |
| Run 2 | Control | Banana | Guava |
| Run 3 | Guava | Control | Banana |
