# Supplementary figures and images for: Exploring the importance and preference of sugar feeding behaviour of malaria vectors in sugar plantations of southern Malawi
Source: PLoS One. 2026 Mar 6;21(3):e0344351. doi: 10.1371/journal.pone.0344351 (PMC12965689; doi:10.1371/journal.pone.0344351)

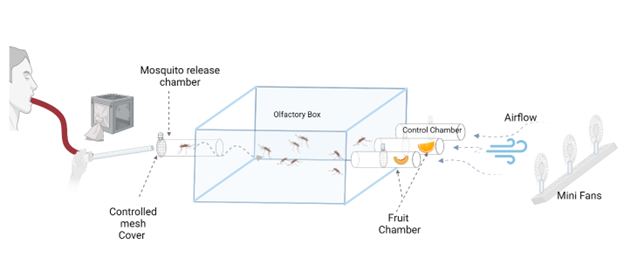

Supplement: S1 File — (TIF) [file pone.0344351.s005.tif]
